# The One-Two Punch of Delirium and Dementia During the COVID-19 Pandemic and Beyond

**DOI:** 10.3389/fneur.2020.596218

**Published:** 2020-11-05

**Authors:** Sara C. LaHue, Vanja C. Douglas, Bruce L. Miller

**Affiliations:** ^1^Department of Neurology, School of Medicine, University of California, San Francisco, San Francisco, CA, United States; ^2^Department of Neurology, Weill Institute for Neurosciences, University of California, San Francisco, San Francisco, CA, United States

**Keywords:** delirium, dementia, Alzheimer's disease and related dementias (ADRD), COVID-19, hospitalization, accessibiity, technology

## Introduction

While the severe acute respiratory syndrome coronavirus 2 (SARS-CoV-2) pandemic is an unprecedented threat to all of us, older adults are especially at risk for serious complications from coronavirus disease 2019 (COVID-19) that necessitate hospitalization (1, 2). Significant attention has rightfully been given to the respiratory and cardiovascular consequences of COVID-19. Less is understood about the neurologic complications associated with this virus, especially its potential impact on delirium and cognitive decline.

The prevalence of delirium is expected to increase during this pandemic due to several factors detailed below (3). Delirium is independently associated with accelerated cognitive decline for those with and without preexisting dementia (4). Adults with Alzheimer's disease and related dementias (ADRD) are particularly vulnerable during this pandemic due to dependence on others and increased likelihood of living in assisted living facilities (5). Further, 30% of all COVID-19 deaths in the United States occur in patients living in nursing homes; a high percentage of these individuals also suffer from mild cognitive impairment or dementia (6). If hospitalized, those with ADRD are more likely to become delirious (7). Our goals are to highlight the heightened risk of this neurologic “one-two punch” as well as provide pragmatic, evidence-based management recommendations for a several clinical environments during the COVID-19 pandemic and subsequent recovery period.

## Recognizing the Hidden Delirium Epidemic Within the Pandemic

Delirium is a common acute disturbance in mental status characterized by fluctuations in attention and cognition, that is more common in older adults and those with dementia (4). Compared with non-delirious patients, hospitalized delirious patients are more likely to develop functional impairment, be discharged to a facility, and be readmitted to the hospital (4, 8). While there are established clinical pathway interventions that modify the risk of developing delirium, there is no drug currently recommended for the prevention or treatment of delirium other than medications needed to treat an identified provoker (e.g., antibiotics for infection) (3, 9). Rather, delirium management centers on non-pharmacologic measures, and the removal of psychoactive medications unless necessary for significant agitation (3). Identifying and managing patients with delirium is paramount as the ability to safely discharge patients home, which is necessary for sustaining strained healthcare systems with limited hospital resources during this pandemic.

While quantifying the impact of this pandemic on delirium is in its early stages, COVID-19 has been demonstrated in small studies to be associated with an increased risk of delirium (10, 11). This association is likely due to an exacerbation of established risk factors, including the development of hypoxia, metabolic derangements, and infection leading to a heightened inflammatory state, as well as the need for intensive care (12, 13). In one point-prevalence cohort study of 71 hospitalized individuals with COVID-19, 31 patients were diagnosed with delirium by DSM-IV criteria (11). Importantly, only 12 of these patients were recognized as being delirious by the primary clinical team (11). Such a discrepancy is not surprising since, without the use of a targeted screening tool, misidentification of delirium for depression or other psychiatric diagnoses, especially in older patients is not uncommon (14, 15). This underdiagnosis of delirium highlights the need for structured clinical pathways with systematic screening to identify delirium so as to appropriately manage these patients (9). Accurate delirium identification is also instrumental for providing informed projections of the long-term resources needed to support those who survive.

One critical outcome of this pandemic is the anticipated exacerbation of delirium in all hospitalized patients, regardless of COVID-19 status (3, 16). This is in large part due to challenges with implementing proper delirium prevention guidelines (3). For example, prior to this pandemic, many hospitals across the United States housed Acute Care for Elders (ACE) Units that incorporated evidence-based practices for delirium prevention spearheaded by the Hospital Elder Life Program (4). Such programs significantly reduced hospital-associated complications for older adults, including delirium, which can be prevented in as many as 40% of cases with these modest interventions (4). However, the management of hospitalized adults has undergone significant systematic changes in response to COVID-19, regardless of COVID-19 status (3, 17). With many hospitals reaching or exceeding patient capacity during this pandemic, some ACE units have disbanded in order to reallocate resources for the care of patients with COVID-19, subsequently fracturing delirium prevention care pathways as well. Furthermore, hospitals have placed restrictions on visitors of hospitalized patients, some prohibiting all visitors regardless of COVID-19 status, with rare exceptions (3, 16, 18). Such restrictions also often apply to caregivers for patients who are delirious or have ADRD, despite playing an essential role in patient care by encouraging physical and cognitive stimulation to prevent delirium, protecting their loved one from falls, and advocating for their basic needs, such as oral hydration (3, 9, 16).

Delirium prevention is even more challenging for hospitalized adults with COVID-19. In addition to strict visitor restrictions, all patients hospitalized for COVID-19 experience heighted solitude and reduced physical activity from quarantine in order to reduce exposure to staff and preserve personal protective equipment (PPE) (16). Sleep may be disrupted if tests occur at night to ensure adequate time for equipment sterilization. While designed to minimize infection risk, these policies also foster a deliriogenic environment (3). Though these unfavorable environmental changes are new, this novel virus has largely incited a resurgence of traditional risk factors for hospital-acquired delirium, and in doing so has exposed fissures in the clinical care of these patients that require urgent, creative solutions.

## Novel Challenges for Adults With Alzheimer's Disease and Related Dementias

Adults with ADRD are especially vulnerable to COVID-19 infection due to their older age, frequency of comorbid conditions such as cardiovascular disease and diabetes, increased reliance on assistance from others for survival, and a higher likelihood of living in assisted living or nursing home, making self-quarantine difficult for many (5, 19). Depending on the severity of the dementia or presence of associated behavioral symptoms, those with ADRD may have difficulty understanding or remembering public health recommendations, such as maintaining good hand hygiene, social distancing, and wearing a face covering (20). Further, in the event that support systems fail—if a family member becomes ill, grocery delivery is not possible, or medication refills delayed—additional harm can result (21). Combined, these challenges may lead to significant psychosocial stressors for those with ADRD and their families during this pandemic, which are only exacerbated in the event of hospitalization (3, 21–23).

Adults with ADRD, especially those diagnosed with COVID-19, are at heightened risk for both hospitalization and delirium during this pandemic (7, 24). While changes to hospital policies, such as restricted visitation and need for isolation, are important for infection control, the consequences of these guidelines are anticipated to be especially detrimental to this population. Even once delirium resolves, its effects can persist, as delirium is associated with an accelerated rate of cognitive decline in those with ADRD (7). Delirium is also an independent risk factor for new cognitive decline in those without pre-existing cognitive impairment (25). In one prospective study, 9.5% of cognitively normal adults with post-operative delirium developed mild cognitive impairment or dementia within a year (25). In addition to the physical and psychological challenges that COVID-19 survivors face, a surge in delirium during this pandemic may lead to a delayed epidemic of cognitive impairment.

## Adapting Evidence-Based Practices for Delirium and ADRD Management

The tenets of delirium prevention are evidence-based and are not antithetical to the precautions needed to care for hospitalized patients, especially those with ADRD, during this pandemic (3). Rather, these recommendations can be adapted for patients with COVID-19, as well as those without COVID-19 but who are still significantly affected by changes in hospital and community policies (Table 1).

**Table 1:** Adapting management practices for hospitalized patients with delirium or dementia during the COVID-19 pandemic.

**Clinical practice adaptations**
Hospital policies:
Allow visitation^a^^,^^b^^,^^c^ for essential caregivers of COVID-19-negative patients with delirium and dementia
Limit non-urgent nighttime disturbances (e.g., testing, room changes)^a^^,^^b^^,^^c^
Screen for cognitive and functional impairments on admission, including visual and hearing impairment; provide assistive devices if needed^a^^,^^b^^,^^c^
Communication and accessibility:
Provide reorientation, including medical staff roles, with each patient encounter^a^^,^^b^^,^^c^
Provide a card with your name, role, and photograph^d^
Offer teleconferencing tools for providers to more readily communicate with patients^e^
Offer teleconferencing tools for patients to easily communicate with their families^f^
Modify communication tools (e.g., voice recognition, automatic call acceptance) as needed
Psychosocial stressors:
Offer teleconferencing access to spiritual/psychological support staff^b^
Limit use of deliriogenic medications for anxiety and mood, such as benzodiazepines^a^^,^^b^^,^^c^
Discharge anticipatory guidance:
May require additional support from family/caregivers^g^ due to overburdened outpatient facilities
Coordination of follow-up with outpatient team,^a^^,^^g^ including training caregiver on use of telehealth tools^e^ prior to discharge
Document delirium diagnosis^g^ and refer to outpatient for follow-up,^a^^,^^b^ including cognitive assessment

a *Hshieh et al. (9)*.

b *Inouye et al. (26)*.

c *Wang et al. (27)*.

d *Arora et al. (28)*.

e *Hatcher-Martin et al. (29)*.

f *Hart et al. (18)*.

g *Khachaturian et al. (30)*.

There are several novel strategies for coping with the isolation from social distancing, largely revolving around technological tools (3, 16). Ideally, the caregivers of hospitalized COVID-19 negative patients with delirium or ADRD would be viewed as essential to patient care and allowed visitation, while still adhering to hospital policies on symptom screening and PPE. If this is not possible, then adapting hospital teleconferencing tools, such as providing electronic tablets, can enhance communication between patients and their families (18). These tools can also increase access to nursing and spiritual/psychological support staff while minimizing infection risk and PPE use.

An important caveat that receives insufficient consideration is that many of these modifications assume abilities on the part of the patient. For instance, those with hearing impairment are at increased risk for delirium and should be provided with assistive devices, such as pocket amplification devices, in order to use teleconferencing tools (9, 26, 27, 31). Recognizing a patient's premorbid cognitive and functional status is important for providing accessible means of communication. Voice recognition software on smartphones and computers allows for placing calls without physically interacting with the device; however, this may not be feasible for individuals with delirium, advanced ADRD or language impairment. One innovative alternative for these individuals is to enable the automatic call accept feature found on many devices, permitting the device to automatically answer telephone calls, or video calls such as FaceTime, from known contacts. This exciting accessibility feature may facilitate more frequent patient contact with family and staff for those who have difficulty interfacing with digital tools. Employing technology creatively to reduce social isolation, provide cognitive stimulation, and more readily assess basic needs in patients with delirium and advanced ADRD is an area that warrants increased attention.

In addition to hospital-specific considerations, the management of adults with ADRD during this pandemic must span the entire continuum of care. Disruption to clinic appointments, even with telemedicine capabilities, will likely result in increased demand for care. Community outpatient access is vital for keeping those with ADRD supported at home and out of the hospital. The rapid expansion of access to teleconferencing tools, including the waiving of Health Insurance Portability and Accountability Act compliance in order to permit more popular video chat applications, is a valuable step (32). Clinical assessments must also be adapted to suit the constraints of digital technologies. For many medical specialties, including several neurologic subspecialities, there is a trend toward non-inferiority of remote assessments compared to in-person visits (17, 29). While it is possible to offer cognitive testing by telemedicine, accuracy may be affected by hearing and vision impairment and so the results of these tests must be interpreted these limitations in mind (33–35). In addition to the special considerations for patients with ADRD, adults diagnosed with hospital-associated delirium should receive outpatient follow-up, including cognitive assessments to screen for cognitive decline. Just as battling this pandemic has been resource-intensive, so too will providing adequate resources to support survivors long-term.

## Conclusions

Delirium is a common complication faced by hospitalized older adults that can often be prevented with modest interventions. Unfortunately, the rate of delirium will likely increase during the COVID-19 pandemic due to both infectious and environmental considerations. While ADRD is a risk factor for delirium, delirium is also an independent risk factor for cognitive decline. If delirium prevention is neglected, and we fail to adapt clinical care to meet these unique challenges, we risk an unprecedented accelerated growth in cognitive impairment in the months and years to come. We cannot afford to let our guard down at this critical juncture.

## Author Contributions

SL made substantial contributions to conception or design of the work, drafted the work, gave final approval of the version to be published, and agreed to be accountable for all aspects of the work. VD and BM made substantial contributions to conception or design of the work, revised the work critically for important intellectual content, gave final approval of the version to be published, and agreed to be accountable for all aspects of the work. All authors contributed to the article and approved the submitted version.

## Conflict of Interest

The authors declare that the research was conducted in the absence of any commercial or financial relationships that could be construed as a potential conflict of interest.
